# Inflammaging reshapes the immune ecosystem in IgG4-related dacryoadenitis and sialadenitis

**DOI:** 10.1093/rheumatology/keag480

**Published:** 2026-09-04

**Authors:** Motohisa Yamamoto, Ryuta Kamekura, Masaaki Uehara, Yuta Ichii, Kenichi Takano

**Affiliations:** Department of Rheumatology, Allergy and Clinical Immunology, IMSUT Hospital, The Institute of Medical Science, The University of Tokyo, Tokyo, Japan; Department of Otolaryngology-Head and Neck Surgery, Sapporo Medical University School of Medicine, Sapporo, Japan; Department of Rheumatology, Allergy and Clinical Immunology, IMSUT Hospital, The Institute of Medical Science, The University of Tokyo, Tokyo, Japan; Department of Rheumatology, Allergy and Clinical Immunology, IMSUT Hospital, The Institute of Medical Science, The University of Tokyo, Tokyo, Japan; Division of Cancer Cell Biology, The Institute of Medical Science, The University of Tokyo, Tokyo, Japan; Department of Otolaryngology-Head and Neck Surgery, Sapporo Medical University School of Medicine, Sapporo, Japan

**Keywords:** IgG4-related disease, inflammaging, immune ecosystem, fibrosis, immunosenescence

## Abstract

**Objectives:**

IgG4-related disease (IgG4-RD) predominantly affects middle-aged and elderly individuals; however, the impact of ageing on lesional immune architecture remains poorly understood.

**Methods:**

Bulk transcriptomic analyses were performed using IgG4-RD lesion tissues across a broad age spectrum. Age-associated genes, Hallmark pathways and immune/stromal module scores were evaluated using correlation-based analyses, network analysis and fibrosis pseudo-staging.

**Results:**

Principal component analysis demonstrated age-associated transcriptomic shifts in IgG4-RD lesions. Ageing was associated with increased expression of cytotoxic/exhaustion-related genes, including IFNG, GZMA, GZMH and PRDM1, together with activation of inflammaging- and senescence-associated programs. Module analysis demonstrated positive correlations between age and fibroblast, exhaustion, macrophage/M2, fibrosis-associated macrophage (FAM), inflammaging and senescence-associated secretory phenotype (SASP) signatures, whereas germinal-centre B cell programs showed minimal association with age. Hallmark pathway analysis revealed enrichment of interferon-γ response, IL6–JAK–STAT3 signalling, TNFα/NFκB signalling, epithelial–mesenchymal transition and TGF-β signalling in ageing lesions. Network analysis demonstrated segregation between adaptive immune modules and ageing-associated fibro-inflammatory modules. Advanced fibrosis pseudo-stages were enriched in older patients.

**Conclusions:**

Ageing in IgG4-RD was associated with coordinated immune ecosystem remodelling characterized by inflammaging, exhaustion-associated immune programs, stromal activation and relative reduction in the prominence of adaptive germinal-centre immunity. These findings suggest that ageing-associated immune remodelling may contribute to chronic fibro-inflammatory disease evolution in IgG4-related dacryoadenitis and sialadenitis.

## Graphical abstract

**Graphical Abstract:**
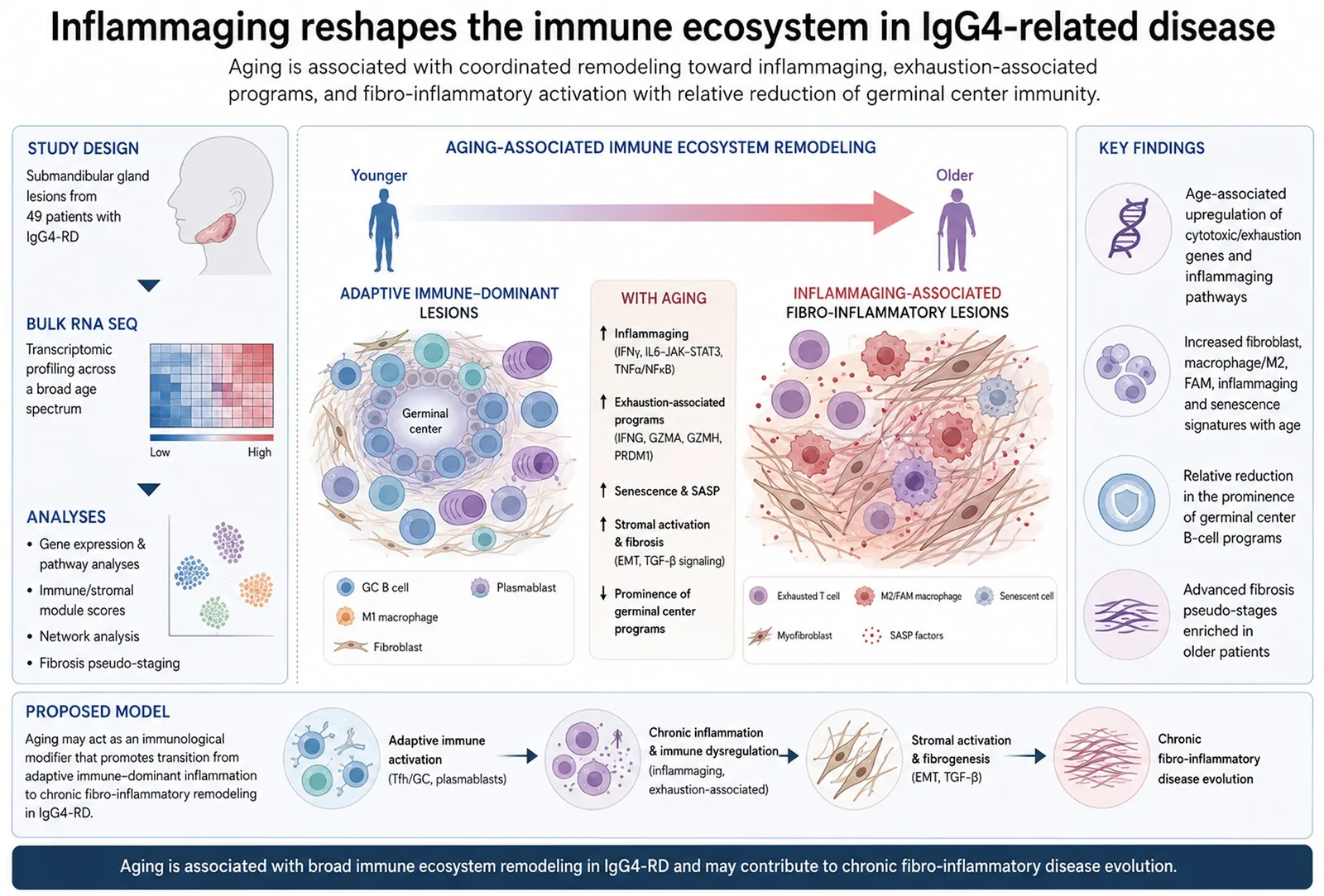

*Rheumatology* key messages

- Ageing in IgG4-RD is associated with remodelling of the lesional immune ecosystem characterized by inflammaging, stromal activation and exhaustion-associated programs.
- Older IgG4-RD lesions exhibit enhanced fibro-inflammatory and senescence-associated immune signatures, whereas germinal-centre-associated programs become relatively less prominent.

## Introduction

IgG4-related disease (IgG4-RD) is a systemic fibro-inflammatory disorder characterized by tumefactive lesions, dense lymphoplasmacytic infiltration, storiform fibrosis and elevated serum IgG4 levels [1, 2]. Clinically, IgG4-RD predominantly affects middle-aged and elderly individuals and has long been recognized as a disease of older adults [1–3]. However, the biological significance of this age predominance has remained largely unclear.

Previous studies have suggested that younger patients with IgG4-RD more frequently exhibit allergic diseases, eosinophilia and elevated serum IgE levels [4], supporting the conventional concept of IgG4-RD as a Th2-associated immune disorder [5, 6]. In contrast, older patients often develop characteristic fibro-inflammatory lesions despite exhibiting fewer allergic manifestations. These clinical observations raise the possibility that ageing itself may influence the immune architecture and pathogenic programs operating within affected tissues. Recent studies have highlighted the importance of tissue immune ecosystems in IgG4-RD, including Tfh/Tph-associated helper T cell responses [7, 8], plasmablast differentiation [9], macrophage-mediated fibro-inflammatory remodelling [10] and ectopic germinal-centre reactions [11]. In parallel, ageing is increasingly recognized as a major driver of chronic immune remodelling through mechanisms such as inflammaging, immune exhaustion, senescence-associated secretory phenotype (SASP) activation and stromal reprogramming [12–15]. Nevertheless, whether these ageing-associated immune programs contribute to lesional immune organization in IgG4-RD remains unknown.

We therefore hypothesized that ageing in IgG4-related dacryoadenitis and sialadenitis is not merely a demographic background factor, but may be associated with broad remodelling of the immune–stromal ecosystem within affected tissues. To address this question, we performed transcriptomic immune ecosystem analyses across a broad age spectrum of IgG4-RD lesions, focusing on age-associated immune programs, inflammaging signatures, adaptive immune remodelling and fibrosis-related tissue evolution.

## Methods

### Subjects and clinical information

Submandibular gland tissues from 49 patients with IgG4-related disease (IgG4-RD) were retrospectively analysed in this study. All tissue samples had been obtained for routine diagnostic evaluation at Sapporo Medical University. All patients fulfilled the 2020 revised comprehensive diagnostic criteria for IgG4-RD [16].

To investigate the relationship between ageing and lesional immune architecture, patients covering a broad age range were included without stratification according to allergic status or fibrosis severity. Clinical information, including age, sex, serum IgG4 levels, serum IgE levels, eosinophil counts, complement levels and allergic comorbidities, was collected from medical records when available.

This study was approved by the Ethics Review Committee of the Institute of Medical Science, The University of Tokyo (Approval No. 2023–22-0720). Written informed consent was obtained from all participants in accordance with the Declaration of Helsinki.

### Transcriptomic profiling and preprocessing

Bulk transcriptomic datasets derived from IgG4-RD submandibular gland lesions were analysed using R software (version 4.5.2 ; R Foundation for Statistical Computing, Vienna, Austria). Gene symbols were arranged in the first column and individual samples in subsequent columns. Duplicate gene symbols were removed by retaining the first occurrence. Expression values were log2-transformed as log2(expression + 1) prior to downstream analyses.

To investigate ageing-associated transcriptomic remodelling, expression values were further standardized across samples for visualization analyses, including heatmaps, principal component analysis (PCA) and ecosystem correlation mapping.

### Ageing-associated immune ecosystem analysis

To characterize the impact of ageing on lesional immune organization, transcriptomic signatures representing adaptive immunity, chronic inflammatory remodelling, senescence-associated programs and stromal activation were analysed.

Immune-related signatures included T follicular helper (Tfh), peripheral helper T cell (Tph), germinal-centre B cell (GC_B), activated B cell, plasmablast, macrophage, M1 macrophage, M2 macrophage, fibrosis-associated macrophage (FAM), CD8 T cell, NK-cell and exhaustion-related programs. In addition, inflammaging and SASP signatures were evaluated as ageing-associated inflammatory programs. Stromal signatures included fibroblast and endothelial programs.

Representative genes included CXCL13, PDCD1, ICOS, IL21 and MAF for Tfh/Tph-related programs; PRDM1, XBP1, MZB1 and JCHAIN for plasmablast programs; CD163, MRC1, SPP1 and CCL18 for macrophage-associated programs and COL1A1, COL3A1, FN1, POSTN and FAP for fibroblast-related remodelling.

For each signature, module scores were calculated as the mean normalized expression value of genes included in the corresponding program. The complete gene lists used for module score calculation are provided in Supplementary Table S1.

### Correlation and pathway analyses

Associations between age and transcriptomic programs were evaluated using Spearman rank correlation analysis. Correlation coefficients and corresponding *P*-values were calculated for individual genes and module scores.

To investigate biological pathways associated with ageing, Hallmark pathway enrichment analysis was performed using gene sets obtained from the Molecular Signatures Database (MSigDB). Genes were ranked according to their correlation with age, and enrichment analyses were performed using correlation-based approaches.

### Ecosystem network analysis

To evaluate global immune ecosystem remodelling associated with ageing, correlation-based network analysis was performed using module scores. Networks were constructed based on Spearman correlation coefficients among immune and stromal programs, and visualized using force-directed layouts.

Adaptive immune programs and ageing-associated inflammatory/stromal programs were evaluated as interconnected ecosystem components rather than isolated pathways.

### Fibrosis pseudo-stage analysis

To investigate the relationship between ageing and fibro-inflammatory progression, fibrosis pseudo-stages were defined according to fibrosis-related transcriptomic signatures, including fibroblast-associated extracellular matrix programs.

Cases were categorized into early, intermediate and advanced fibrosis pseudo-stage groups according to relative enrichment of fibrosis-related transcriptomic signatures derived from fibroblast-associated extracellular matrix genes, including COL1A1, COL3A1, FN1, POSTN and FAP. Pseudo-stage classification was performed using tertile stratification of fibrosis module scores across all samples. Age distributions across pseudo-stage groups were subsequently compared.

### Statistical analysis

All statistical analyses were performed using R software (version 4.5.2). Correlation analyses were conducted using Spearman rank correlation coefficients. Group comparisons were performed using non-parametric statistical methods where appropriate. All statistical tests were two-sided, and *P*-values <0.05 were considered statistically significant.

## Results

### Clinical characteristics and age-associated transcriptomic heterogeneity in IgG4-RD

A total of 49 patients with IgG4-RD were included in this study, covering a broad age spectrum ranging from the fourth to eighth decades of life (Fig. 1A). Clinical laboratory parameters, including serum IgG4, IgG, IgE, eosinophil counts and complement activity, demonstrated substantial inter-individual heterogeneity across patients (Table 1).

**Figure 1:**
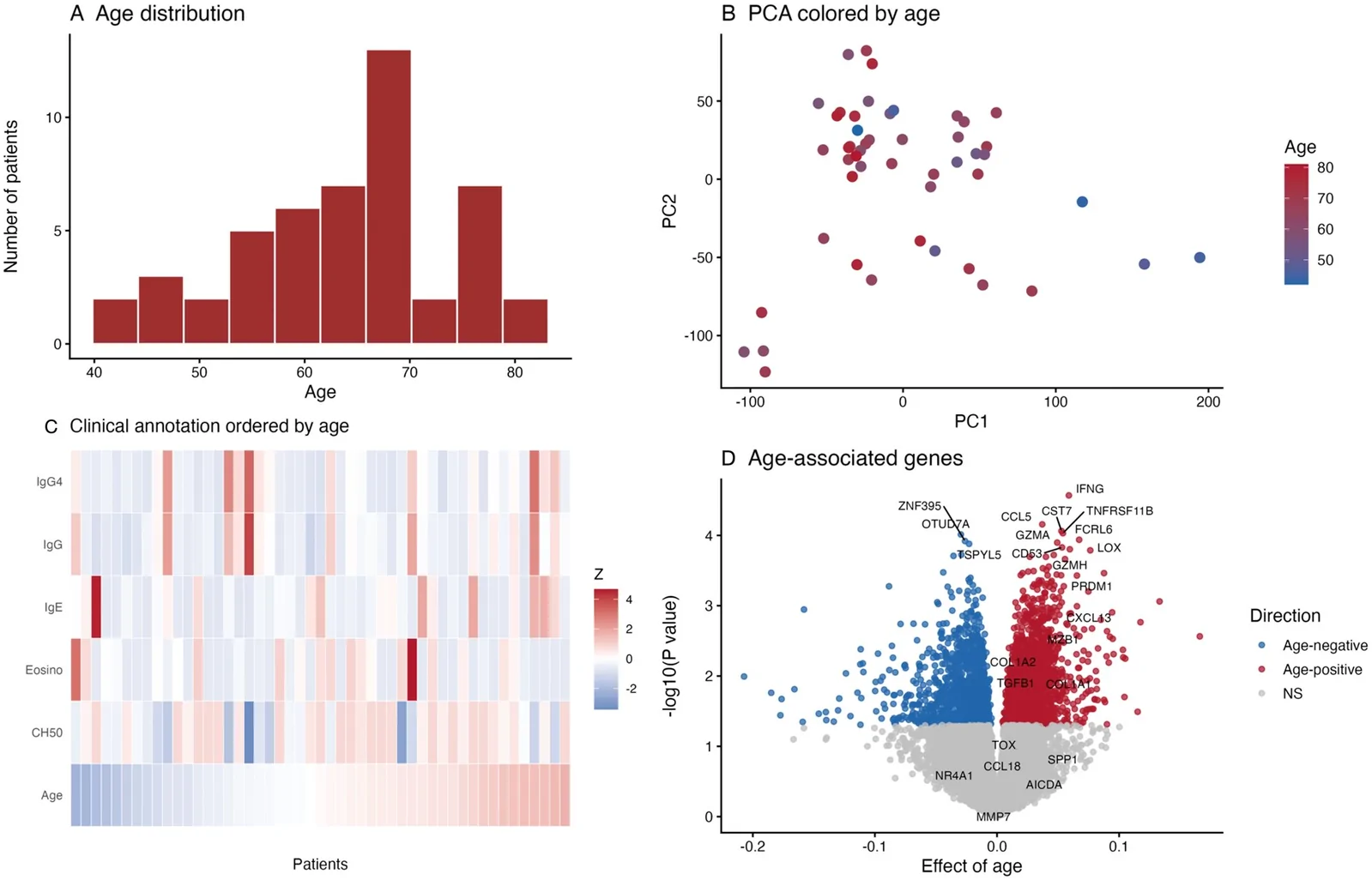
Age-associated transcriptomic landscape in IgG4-related disease lesions. (**A**) Distribution of patient age in the analysed IgG4-RD cohort. (**B**) Principal component analysis (PCA) of bulk transcriptomic profiles coloured according to patient age, demonstrating a partial age-associated shift in global gene expression patterns. (**C**) Clinical annotation heatmap ordered by age. Clinical parameters including serum IgG4, IgG, IgE, eosinophil count and CH50 are shown as *Z*-score normalized values. (**D**) Volcano plot showing genes associated with ageing in IgG4-RD lesions. Genes positively associated with age, genes negatively associated with age and non-significant genes are indicated separately. Ageing was associated with increased expression of cytotoxic/exhaustion-related immune genes (e.g., IFNG, GZMA, GZMH, CCL5 and PRDM1) and fibrosis-associated stromal genes (e.g., COL1A1, COL1A2, TGFB1 and LOX), suggesting age-related remodelling of the immune–stromal ecosystem in IgG4-RD lesions

**Table 1:** Clinical characteristics of patients with IgG4-related disease and healthy controls.

Variable	All patients (*n* = 49)
Age, years	65.0 (58.0–70.0)
Male sex, *n* (%)	22 (44.9%)
Serum IgG, mg/dl	1776.0 (1484.0–2320.0)
Serum IgG4, mg/dl	387.0 (251.0–785.0)
Serum IgE, IU/ml	256.0 (93.0–680.0)
CH50	53.7 (42.5–60.0)
Eosinophils, /μl	30.0 (0.0–173.0)

Data are presented as median (interquartile range) unless otherwise indicated.

PCA demonstrated partial age-associated segregation of transcriptomic profiles, suggesting that ageing contributes to global remodelling of lesional gene expression programs in IgG4-RD (Fig. 1B). Clinical annotation heatmaps further demonstrated that conventional laboratory parameters did not show clear age-dependent clustering patterns despite progressive increases in patient age (Fig. 1C), indicating that transcriptomic ageing-related remodelling may not be fully captured by routine clinical biomarkers.

To identify ageing-associated genes, correlation analyses between age and transcriptomic expression profiles were performed. Ageing was positively associated with cytotoxic and exhaustion-related genes, including IFNG, GZMA, GZMH, PRDM1, TNFRSF11B and CXCL13, together with fibrosis-associated genes such as COL1A1, COL1A2, TGFB1 and LOX (Fig. 1D). In contrast, several adaptive immune-associated genes, including NR4A1 and AICDA, showed relatively reduced expression with ageing.

### Ageing is associated with fibro-inflammatory and exhaustion-related immune ecosystem remodelling

To investigate how ageing influences lesional immune architecture, immune and stromal transcriptomic modules were analysed. Among analysed programs, fibroblast and exhaustion signatures demonstrated the strongest positive correlations with age, followed by macrophage, plasmablast, M2 macrophage, Tph, fibrosis-associated macrophage (FAM), inflammaging and SASP programs (Fig. 2A). In contrast, GC_B signatures demonstrated minimal association with ageing.

**Figure 2:**
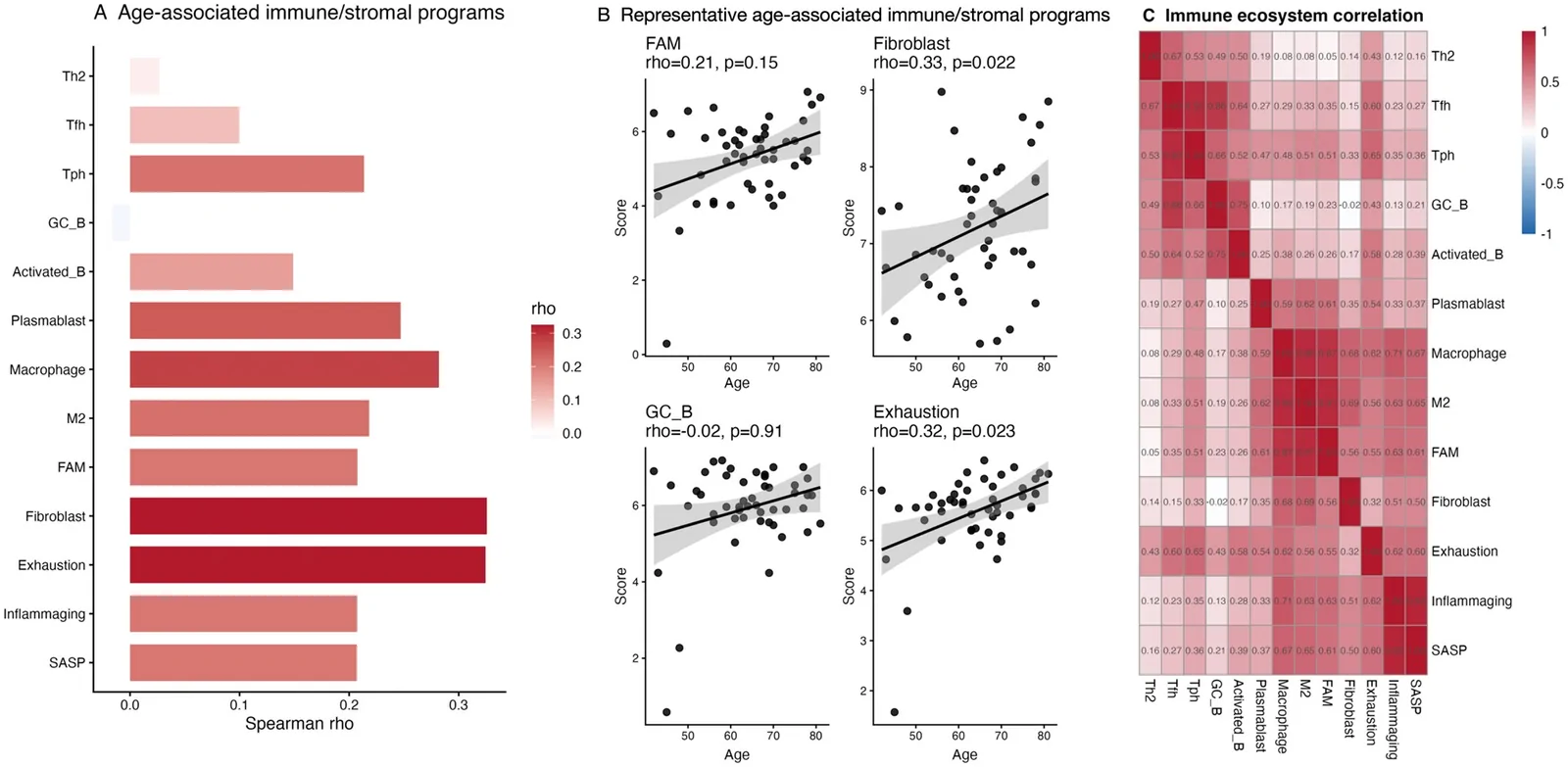
Ageing-associated remodelling of immune and stromal programs in IgG4-related disease. (**A**) Spearman correlation analysis between patient age and immune/stromal module scores. Ageing was positively associated with Tph, plasmablast, macrophage, M2 macrophage, fibrosis-associated macrophage (FAM), fibroblast, exhaustion, inflammaging and senescence-associated secretory phenotype (SASP) programs, whereas GC_B and Th2 programs showed minimal association with age. (**B**) Representative scatter plots demonstrating the relationship between age and selected module scores, including FAM, fibroblast, GC_B and exhaustion signatures. Regression lines with 95% CIs are shown. (**C**) Correlation heatmap of immune and stromal ecosystem modules. Strong correlations were observed among macrophage/M2/FAM programs, fibroblast activation, exhaustion, inflammaging and SASP signatures, suggesting coordinated age-associated remodelling of the fibro-inflammatory immune ecosystem in IgG4-RD lesions

Representative analyses demonstrated significant positive correlations between age and fibroblast as well as exhaustion-related programs, whereas GC_B programs remained largely unchanged across the age spectrum (Fig. 2B). Correlation matrix analysis revealed segregation between adaptive immune programs and ageing-associated fibro-inflammatory modules, with strong interconnections among macrophage, M2, FAM, fibroblast, inflammaging and SASP signatures (Fig. 2C).

Comprehensive analyses across all evaluated modules confirmed that fibroblast, exhaustion, macrophage and plasmablast-associated programs consistently demonstrated the strongest ageing-associated enrichment (Supplementary Fig. S1).

### Ageing lesions exhibit inflammaging and senescence-associated pathway activation

To further characterize biological pathways associated with ageing in IgG4-RD lesions, Hallmark pathway enrichment analyses were performed. Ageing was strongly associated with enrichment of interferon-γ response, inflammatory response, IL6–JAK–STAT3 signalling, TNFα/NFκB signalling, epithelial–mesenchymal transition, complement activation and TGF-β signalling pathways (Fig. 3A).

**Figure 3:**
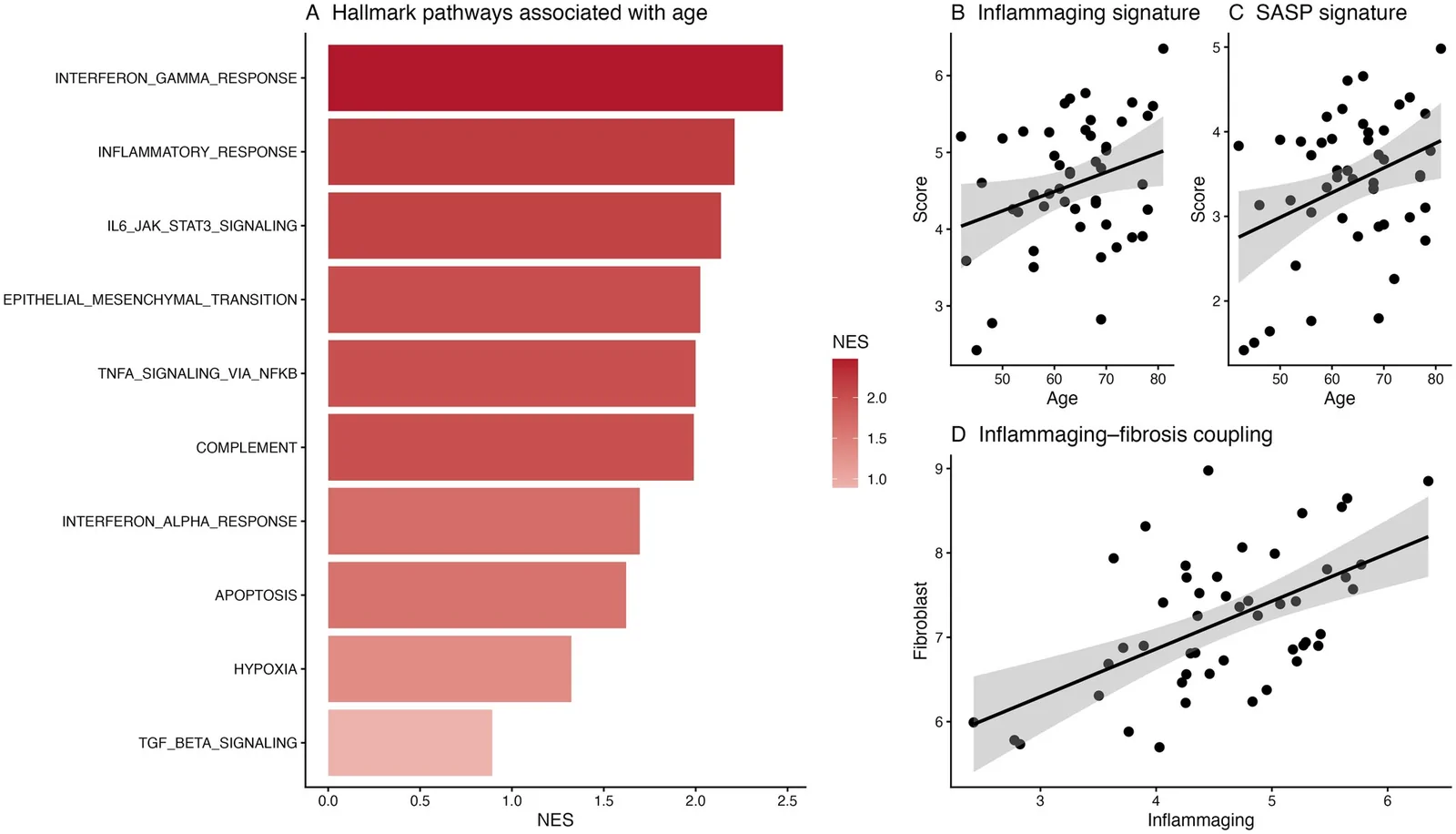
Ageing-associated inflammatory and fibrotic pathway activation in IgG4-related disease. (**A**) Hallmark pathway enrichment analysis associated with age in IgG4-RD lesions. Ageing was positively associated with pathways related to interferon-γ response, inflammatory response, IL6–JAK–STAT3 signalling, epithelial–mesenchymal transition (EMT), TNFα signalling via NFκB, complement activation, hypoxia, apoptosis and TGF-β signalling. Bars indicate normalized enrichment scores (NES). (**B**) Scatter plot showing the association between patient age and inflammaging signature score. (**C**) Scatter plot demonstrating the positive association between age and senescence-associated secretory phenotype (SASP) signature score. (**D**) Correlation between inflammaging and fibroblast signatures, demonstrating coupling between ageing-associated inflammatory programs and stromal/fibrotic remodelling in IgG4-RD lesions. Regression lines with 95% CIs are shown

Because inflammaging and senescence-associated inflammatory remodelling emerged as prominent ageing-associated features, we next evaluated these programs in detail. Both inflammaging and SASP signatures demonstrated progressive increases with age (Fig. 3B and C). Furthermore, inflammaging signatures strongly correlated with fibroblast-associated remodelling, suggesting coordinated coupling between chronic inflammatory ageing programs and tissue fibrosis (Fig. 3D).

Despite these transcriptomic alterations, conventional clinical biomarkers, including serum IgG4, IgG, IgE, CH50 and eosinophil counts, demonstrated only weak or minimal associations with ageing (Supplementary Fig. S2), indicating that ageing-associated immune remodelling may occur independently of routine serological disease activity markers.

### Ageing-associated immune ecosystem transition accompanies fibro-inflammatory progression

To investigate the relationship between ageing and disease evolution, fibrosis pseudo-stage analyses were performed. Advanced fibrosis pseudo-stage lesions were enriched in older patients, whereas younger patients more frequently exhibited early-stage lesions (Fig. 4A).

**Figure 4:**
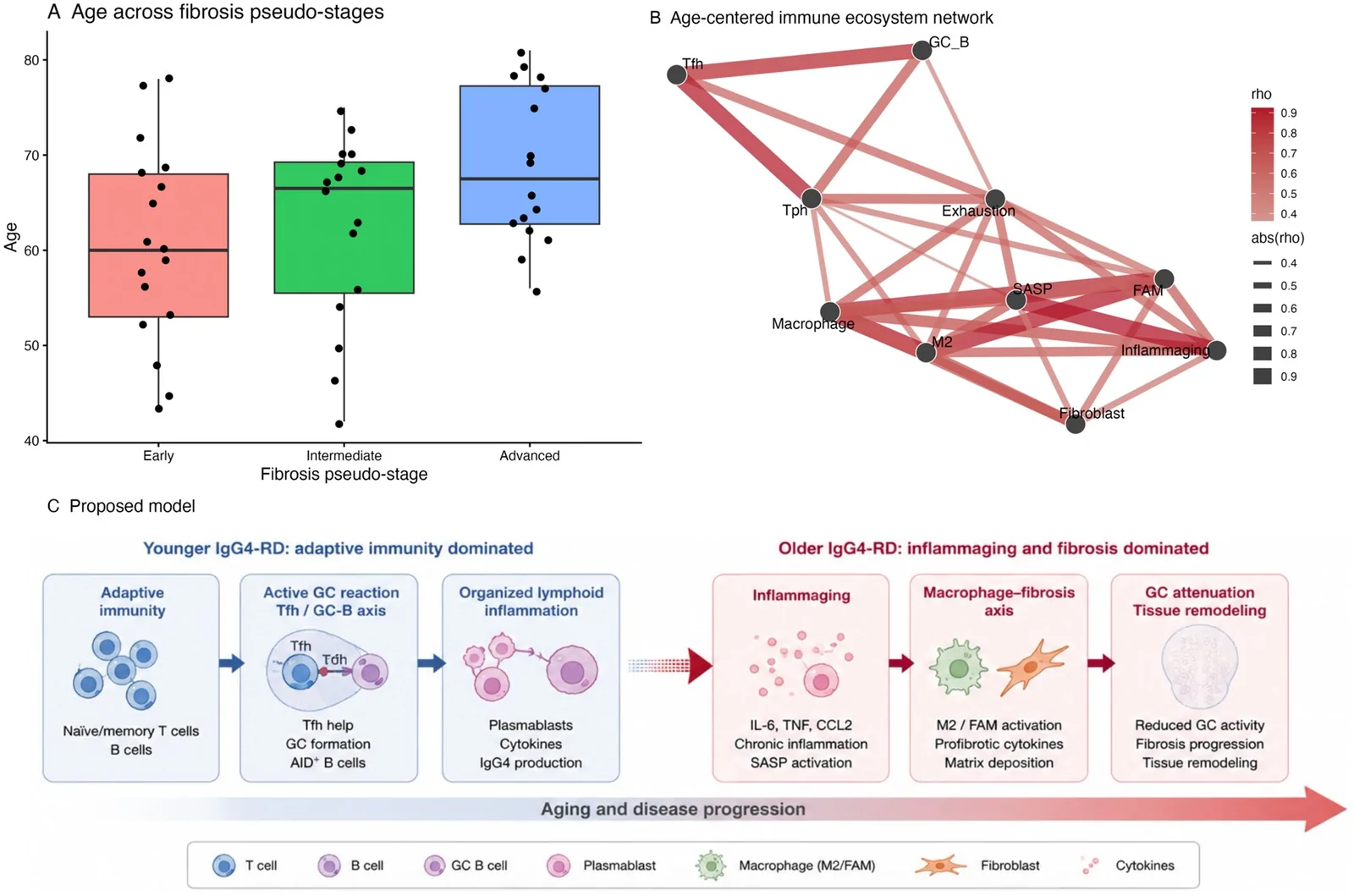
Proposed model of ageing-associated immune ecosystem remodelling in IgG4-related disease. (**A**) Patient age across fibrosis pseudo-stages. Cases classified into advanced fibrosis pseudo-stages tended to exhibit older age distributions compared with early-stage lesions, suggesting an association between ageing and fibro-inflammatory progression. (**B**) Age-centered immune ecosystem network analysis. Correlation-based network visualization demonstrated segregation between adaptive immune modules (Tfh and GC_B) and ageing-associated fibro-inflammatory modules, including macrophage/M2, fibrosis-associated macrophages (FAM), fibroblast activation, inflammaging and SASP signatures. Tph and exhaustion programs occupied intermediate network positions linking adaptive immunity and chronic inflammatory remodelling. Edge thickness and colour intensity represent correlation strength. (**C**) Proposed model of ageing-associated disease evolution in IgG4-RD. Younger lesions are characterized by adaptive immunity–dominant inflammation with active Tfh/GC_B interactions and organized lymphoid responses. In contrast, older lesions exhibit inflammaging, SASP activation, macrophage–fibrosis coupling, fibroblast activation and relative reduction in germinal-centre-associated immune programs, ultimately leading to chronic fibro-inflammatory tissue remodelling

Network analysis demonstrated that adaptive immune modules, including Tfh, Tph and GC_B programs, occupied positions distinct from ageing-associated fibro-inflammatory programs such as inflammaging, SASP, macrophage/FAM, exhaustion and fibroblast modules (Fig. 4B). Notably, Tph and exhaustion programs occupied intermediate positions linking adaptive immune responses and chronic fibro-inflammatory remodelling.

Based on these findings, we propose a model in which younger IgG4-RD lesions are characterized by adaptive immune-dominant responses with active Tfh/GC_B interactions and organized lymphoid inflammation, whereas older lesions progressively shift towards inflammaging-associated fibro-inflammatory remodelling characterized by macrophage activation, stromal remodelling, exhaustion-associated programs and attenuation of germinal-centre immunity (Fig. 4C).

## Discussion

In this study, we identified ageing-associated immune ecosystem remodelling as a central biological feature of IgG4-related disease (IgG4-RD). Rather than simply reflecting cumulative fibrosis or disease duration, ageing was associated with coordinated alterations in lesional immune architecture characterized by inflammaging, senescence-associated inflammatory programs, exhaustion-associated immune signatures, stromal activation and relative reduction in the prominence of germinal-centre-associated immune programs within the ageing immune ecosystem. These findings suggest that ageing is associated with broad remodelling of the lesional immune ecosystem in IgG4-RD and may contribute to chronic fibro-inflammatory disease evolution. IgG4-RD has traditionally been regarded as a Th2-associated fibro-inflammatory disorder linked to allergic manifestations, eosinophilia and elevated serum IgE levels [4–6]. However, this conventional framework does not fully explain the clinical heterogeneity observed across age groups. Younger patients frequently exhibit allergic backgrounds and eosinophilic inflammation [4], whereas older patients often develop progressive fibro-inflammatory organ disease despite relatively limited allergic manifestations. The biological significance of this age-dependent clinical contrast has remained unclear for many years. Our findings suggest that these differences may reflect ageing-associated reorganization of the lesional immune ecosystem itself.

One of the most striking observations in this study was the emergence of inflammaging- and senescence-associated programs in ageing lesions. Older lesions demonstrated enrichment of IFNγ response, IL6–JAK–STAT3 signalling, TNFα/NFκB signalling and chronic inflammatory remodelling pathways. This ageing-associated inflammatory shift was accompanied by enrichment of cytotoxic and exhaustion-related programs, including IFNG- and granzyme-associated signatures, potentially linking ageing lesions to chronic cytotoxic immune remodelling previously implicated in IgG4-RD tissue injury [17]. Although these transcriptomic programs were interpreted as exhaustion-associated signatures, bulk transcriptomic analyses cannot fully distinguish chronic T cell exhaustion from sustained cytotoxic activation states. Therefore, these findings should be interpreted as exhaustion-associated immune remodelling rather than definitive evidence of terminal T cell exhaustion.

These findings are consistent with the concept that ageing promotes persistent low-grade inflammatory activation through inflammaging and immune senescence [12–15]. Importantly, these alterations were observed more clearly at the tissue transcriptomic ecosystem level than in conventional serological biomarkers, suggesting that ageing-associated disease evolution in IgG4-RD may not be adequately captured by routine clinical parameters alone.

Another important finding was the relative attenuation of germinal-centre-associated programs in ageing lesions. Although Tfh/Tph-related helper T cell signatures remained detectable across age groups [7, 8], fibro-inflammatory, senescence-associated and exhaustion-related programs became increasingly dominant with ageing. This pattern suggests that ageing in IgG4-RD is not simply associated with progressive fibrosis, but rather with a broader shift in lesional immune state—from organized adaptive germinal-centre-oriented immunity towards chronic inflammaging-associated innate/stromal remodelling. In this context, younger lesions may preferentially maintain active adaptive immune interactions characterized by Tfh/GC_B responses and plasmablast differentiation [7–9], whereas older lesions progressively acquire features of immune senescence, chronic tissue remodelling and fibro-inflammatory persistence.

Although inflammaging and the SASP are well-established hallmarks of physiological ageing observed across multiple tissues, our findings suggest that these age-associated programs are not merely passive consequences of ageing in IgG4-related dacryoadenitis and sialadenitis. Rather, inflammaging was accompanied by coordinated enrichment of plasmablast-, macrophage-, fibroblast- and exhaustion-associated immune programs together with relative attenuation of GC_B signatures. These coordinated alterations indicate remodelling of the local immune ecosystem rather than a non-specific age-related tissue response. We therefore propose that ageing provides a permissive inflammatory milieu that becomes integrated into the disease-specific immune architecture, thereby promoting chronic fibro-inflammatory tissue remodelling.

The network analyses further support this interpretation. Adaptive immune modules and ageing-associated fibro-inflammatory modules formed relatively distinct ecosystem clusters, while Tph and exhaustion-related programs occupied intermediate positions linking these states [7, 8]. These findings raise the possibility that Tph/exhaustion-associated immune programs may represent transitional states between adaptive immune activation and chronic fibro-inflammatory remodelling in ageing lesions. Although this interpretation remains speculative, it provides a potential conceptual framework connecting chronic helper T cell activation, immune senescence and stromal remodelling in IgG4-RD.

Importantly, our proposed model does not merely represent a fibrosis progression model. Rather, the present findings support the concept of an ageing-associated disease-state transition in IgG4-RD. In this framework, ageing may function as an immunological modifier that alters the balance between adaptive immunity and chronic fibro-inflammatory remodelling within affected tissues.

Several limitations should be acknowledged. First, this was a retrospective transcriptomic study with a relatively limited sample size. Second, bulk RNA-seq analyses cannot fully resolve cell type-specific ageing-associated alterations. Third, because this study focused exclusively on salivary gland lesions from patients with IgG4-related dacryoadenitis and sialadenitis, further investigation will be required to determine whether similar ageing-associated immune ecosystem remodelling occurs in other organ manifestations of IgG4-RD. Integration of spatial transcriptomics and single-cell approaches may provide deeper insights into the cellular architecture underlying inflammaging-associated tissue remodelling. In addition, because fibrosis severity itself may influence tissue immune and stromal transcriptomic composition, the ageing-associated remodelling observed in this study may partially reflect cumulative fibro-inflammatory tissue evolution. Further longitudinal and spatial studies will be required to distinguish ageing-specific effects from fibrosis-dependent remodelling.

In conclusion, our findings suggest that ageing in IgG4-related dacryoadenitis and sialadenitis is associated with broad immune ecosystem remodelling characterized by increased inflammaging-associated fibro-inflammatory programs and relative reduction in the prominence of adaptive germinal-centre immunity. These findings provide a conceptual framework linking ageing biology, chronic immune remodelling and fibro-inflammatory tissue evolution in IgG4-RD. Collectively, these findings support the concept that ageing functions not simply as a background demographic factor, but as an immunological modifier that becomes integrated into the disease-specific immune ecosystem and promotes chronic fibro-inflammatory remodelling.

## Supplementary Material

keag480_Supplementary_Data

## Data Availability

The data underlying this article will be shared on reasonable request to the corresponding author.
