# Supplementary material for: Inflammaging reshapes the immune ecosystem in IgG4-related dacryoadenitis and sialadenitis
Source: Rheumatology (Oxford). 2026 Sep 4;65(10):keag480. doi: 10.1093/rheumatology/keag480 (PMC13641169; doi:10.1093/rheumatology/keag480)
Supplement: Supplementary file 1 [file keag480_supplementary_data.docx]

**Supplementary Figure S1. Correlation of all immune/stromal modules with age in IgG4-RD**

**Supplementary Figure S2. Relationship between age and conventional clinical biomarkers in IgG4-RD**

**Supplementary Table S1. Gene signatures used for immune and stromal module analyses**

| **Module** | **Genes used for module score calculation** |
| --- | --- |
| Tfh | CXCR5, PDCD1, BCL6, ICOS, IL21 |
| preTfh | PDCD1, ICOS, IL21, MAF, SLAMF6, CD200 |
| Tph | PDCD1, CXCL13, IL21, MAF, ICOS |
| Th1 | TBX21, CXCR3, STAT1, IFNG |
| Th2 | GATA3, IL4R, IL13, PTGDR2 |
| Th17 | RORC, CCR6, IL17A, IL23R |
| Treg | FOXP3, IL2RA, CTLA4, TIGIT |
| CD4_CTL | GZMB, GZMA, GZMK, PRF1, NKG7, IFNG, CX3CR1, KLRG1, TBX21, EOMES |
| CD8_T | CD8A, CD8B, GZMB, PRF1, NKG7 |
| NK | NKG7, GNLY, KLRD1 |
| GC_B | MS4A1, CD79A, CD79B, BCL6, AICDA, CD38 |
| Activated_B | CD79A, CD79B, HLA-DRA, CD74, BANK1 |
| Extrafollicular_B | TBX21, ITGAX, FCRL5, CD11C, BLIMP1 |
| Plasmablast | PRDM1, XBP1, MZB1, JCHAIN |
| Macrophage | CD68, CD163, CSF1R, LST1, AIF1 |
| M1 macrophage | IL1B, TNF, CXCL9, CXCL10, NOS2 |
| M2 macrophage | CD163, MRC1, MSR1, IL10, TGFB1 |
| Fibrosis-associated macrophage (FAM) | CD163, MRC1, TGFB1, SPP1, CCL18 |
| cDC1 | CLEC9A, XCR1, BATF3, IRF8, THBD, CADM1, ID2 |
| cDC2 | CD1C, FCER1A, CLEC10A, CD1E, IRF4, KLF4, SIRPA |
| FDC | CXCL13, CR2, FDCSP, FCER2 |
| Fibroblast | COL1A1, COL3A1, FN1, POSTN, FAP |
| Endothelial | PECAM1, VWF, CDH5, KDR |
| Exhaustion-associated program | PDCD1, HAVCR2, LAG3, TIGIT, TOX, NR4A1, IFNG, GZMB |
| Inflammaging | IL6, TNF, CXCL8, CCL2, IFNG, NFKB1 |
| SASP | IL6, CXCL8, MMP3, MMP9, TGFB1, SERPINE1 |

**Abbreviations:** Tfh, T follicular helper cell; Tph, peripheral helper T cell; GC_B, germinal center B cell; FAM, fibrosis-associated macrophage; SASP, senescence-associated secretory phenotype; FDC, follicular dendritic cell.
